# Opioid Agents and Cardiac Arrhythmia: A Literature Review

**DOI:** 10.7759/cureus.38007

**Published:** 2023-04-23

**Authors:** Azamatov Golibkhon, Bazarbaev Akbar Gafur Ugli, Muzaffar Makhamadjonov Farkhod Ugli

**Affiliations:** 1 Department of General Internal Medicine, Almalyk City Central Family Outpatient Hospital, Almalyk, UZB; 2 Department of Medicine, Tashkent Medical Academy, Tashkent, UZB; 3 Department of General Internal Medicine, Tashkent City Family Outpatient Hospital number 43, Tashkent, UZB

**Keywords:** atrial arrhythmias., qt interval, arrhythmogenicity, chronic pain, opioids, cardiac arrythmia

## Abstract

Opioids are compounds that cause similar effects to morphine by binding to its receptors. Opioids can be synthetic, semi-synthetic, or natural and can easily bind to the receptors of opioids in order to depict their effects, which may vary depending upon the exposure of the drug and its dose. However, several side effects of opioids can also be observed, with the most crucial being their impact on the heart’s electrical activity. This review majorly focuses on opioids' impact on the prolongation of the QT curve and their arrhythmogenic susceptibility. Articles published up to the year 2022 in various databases were identified and searched with the use of keywords. Search terms included “cardiac arrhythmias,” “QT interval,” “opioids,” “opioid dependence,” and “torsade de pointes (TdP)”. These terms highlight the impact of each opioid agent on the activity of the heart on an electrocardiogram. The results of the available data depict that opioids, such as methadone, pose higher risks, even when taken in smaller amounts, and have the capability for QT interval prolongation and TdP development. A variety of opioids, i.e., oxycodone and tramadol, are considered as intermediary risk drugs and can build long QT intervals and TdP in large doses. Several other opioids are considered low-risk drugs, including buprenorphine and morphine, which lead to no production of TdP and QT interval prolongation in daily routine doses. Evidence indicates a high risk of sinus bradycardia, atrial fibrillation, cardiac block, and supra-ventricular arrhythmias in opium consumers. This literature review will play a key role in determining the association between the use of opioids and cardiac arrhythmias. It will further highlight the practical implications of opioids for the management of cardiac issues based on their dose, frequency, and intensity. Moreover, it will also depict the adverse effects of opioids along with their dose-specific relationship. Opioids display disparate cardiac arrhythmogenicity, and methadone contains a greater ability to induce long QT intervals and hazardous arrhythmias at conventional doses. In order to reduce arrhythmogenic risk, opioids taken in large amounts should be monitored with a regular electrocardiogram in high-risk consumers, i.e., patients on opioid maintenance.

## Introduction and background

Opioids are drugs used for the relief of pain [1]. They are sensitive to opioid-specific receptors, which are present in the central nervous system [2]. Morphine, being an active painkiller, is derived directly from opium, whereas heroin, being diamorphine, is derived semi-synthetically from morphine [3]. Opium has been isolated from crude substances and contains morphine, thebaine, and codeine [2]. It has been observed that the use of opioids for medical purposes has increased globally; however, their use is only restricted to the treatment of cancer [4]. With time, their use has been extended toward the use of pain relief for non-cancer patients and the management of patients suffering from chronic pain [5]. The utilization of opioids for the management of chronic pain has come under scrutiny for being associated with the risks of cancer and long-term management [6]. Some opioids are synthetic, i.e., pethidine and methadone, while others are endogenous opioids that are produced in the body, such as endorphins. The global opioid consumption rate is on the rise [7]. The pharmacological uses of the drug include opioid use replacement therapy, suppression of cough, diarrhea, and opioid overdose reversal [8,9]. This study aims to lower the drug-related death risk, criminal activity incidence, the transmission of HIV and hepatitis C and B, use of illicit drugs, and episodes of drug dissipation duration by enhancing overall family, social, and personal functioning to accommodate public health [10].

The receptors of opioids are present in all systems, including the gastrointestinal, peripheral, and central nervous systems, to mediate the somatic and psychoactive effects of opioids [11,12]. The drug maintains its physiological and pharmacological activities mainly between three considerable opioid receptor classes: kappa, delta, and mu. Mu agonists, including buprenorphine, methadone, and morphine, are the most commonly used opioids in treating chronic pain and opioid maintenance treatment (OMT) [13]. Opioids alone do not depress the contractility of the heart until used in combination with meperidine [10]. If contractility is not affected by opioids, then they may affect other functions of the cardiovascular system [14,15]. If opioids are administered in acute cases, then it may result in a reduction in sympathetic tone along with vasodilation [16]. In combined administration with benzodiazepines, opioids may affect the output of the heart [17]. The adverse effects of opioids include constipation, respiratory depression, nausea, headache, vomiting, disturbance of electrical activity of the heart, bradycardia, dysrhythmias, and lower cardiac output [18]. The most common side effect of opioids is QT prolongation, which can lead to torsade de pointes (TdP), a type of ventricular tachyarrhythmia that may lead to sudden death [6]. In addition, the FDA has recommended investigating all new drugs with systemic bioavailability for their effect on QT prolongation before their approval [19].

The pharmacodynamics of opioids is dependent upon the binding receptors, including receptor affinity and the quality of the receptor, i.e., being either an agonist or antagonist [20]. Opioids display supraspinal analgesic effects in the form of the agonist morphine, which is mediated by μ1 receptor activation. Physical and respiratory depressions are mediated by the activation of μ2 receptors [21,22]. Opioids have demonstrated quite minimal effects on the vasomotor control of the coronary vessels [20,23]. Studies have depicted that the use of opioids leads to an increase in the risk of cardiovascular events, including cardiac arrhythmias, infarcted myocardium, and failure of the heart [24,25]. However, meager literature suggesting the association of long-term effects on cardiovascular diseases exists [26-32].

## Review

The data related to this research paper has been collected from various databases, such as Google Scholar, PubMed, and the Cumulative Index to Nursing and Allied Health Literature (CINAHL). All papers containing the 20 opioid names, i.e., “tramadol,” “nuprenorphine,” “morphine,” “loperamide,” “oxymorphone,” “methadone,” “pethidine,” “hydrocodone,” “oxycodone,” “codeine,” “hydromorphone,” “levo alpha-acetylmethadol (LAAM),” “tapentadol,” “levorphanol,” “fentanyl and its analogs”, as well as “corrected QT interval (QTc),” “QT interval,” “ventricular arrhythmias,” “QT prolongation,” “the human ether-à-go-go-related gene (hERG),” “atrial arrhythmias,” “opium” and “TdP” were identified and searched. Each paper was shielded for pertinent information on arrhythmogenicity and opioids' adverse effects on the electrocardiogram (ECG).

**Figure 1:**
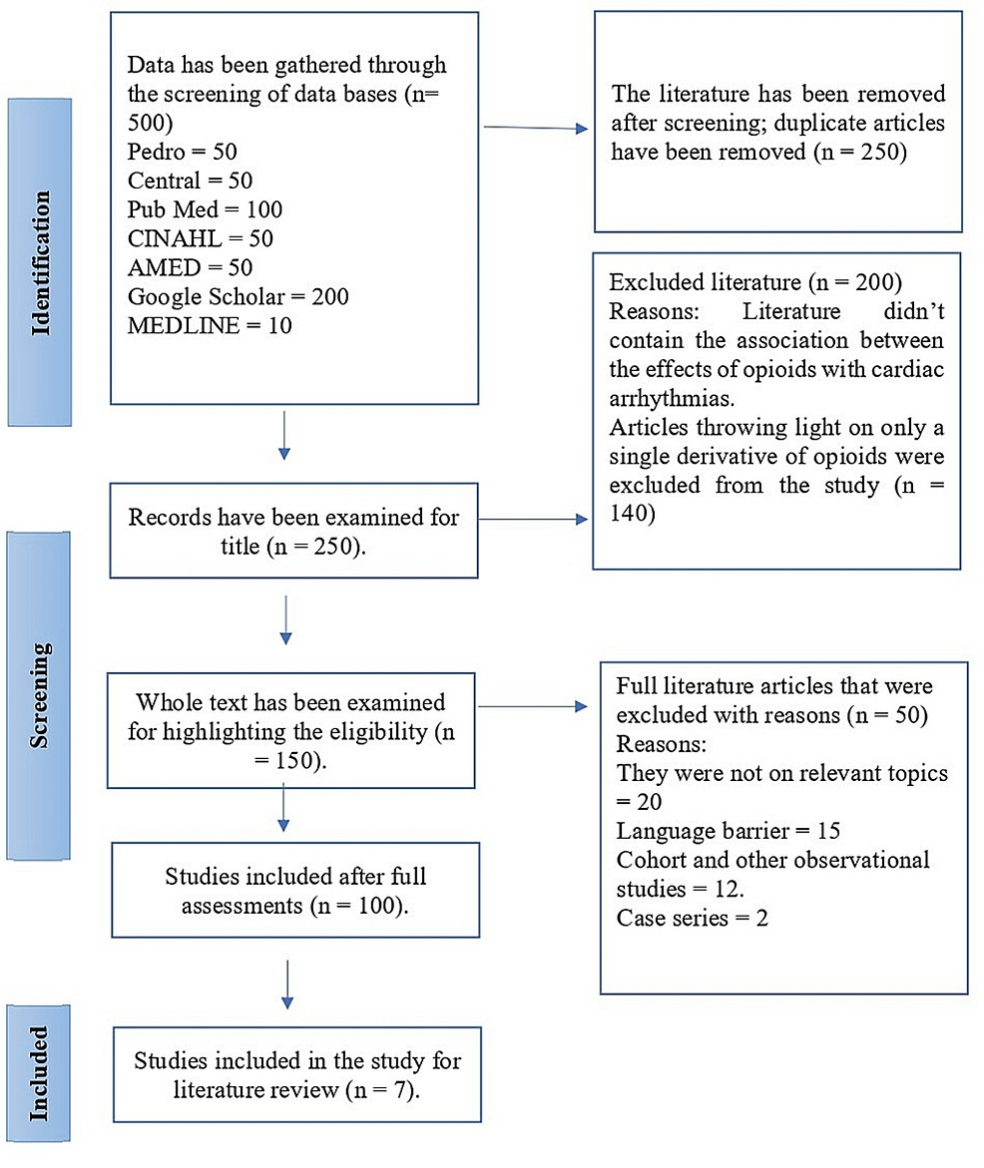
PRISMA flow chart PRISMA: Preferred Reporting Items for Systematic Reviews and Meta-Analyses; CINAHL: Cumulative Index to Nursing and Allied Health Literature; MEDLINE: Medical Literature Analysis and Retrieval System Online; AMED: Allied and Complementary Medicine Database

To perform a literature review, an electronic search was carried out between the years 2022 and 2023. The initial strategy was executed through the use of registered databases, such as the international prospective register of systematic reviews (PROSPERO) and Google Scholar. No previous literature reviews were found on the topic under discussion. Further searches were carried out by making use of databases such as Physiotherapy Evidence Database (PEDro), CINAHL, Medical Literature Analysis and Retrieval System Online (MEDLINE), PubMed, and Google Scholar. These databases were selected to specifically search for scientific literature. Some of these databases were easily accessible, whereas others were only accessible through the University library. A search in the MEDLINE database was carried out by using an asterisk (*) symbol in the searches. The symbol was placed at the end of the words for the substitution during literature searching. A literature review was carried out using Preferred Reporting Items for Systematic Reviews and Meta-Analyses (PRISMA) guidelines. (Figure 1)

Different keywords were used to search the articles in the databases, including “opioids,” “cardiac arrhythmias,” “atrial fibrillation,” and “heart diseases”. The search was enhanced and limited by using the Boolean operators ‘OR’ and ‘AND’. Inclusion criteria involved literature published in the English language that discussed the association of opioids with cardiac arrhythmia. All the recent literature between the period of 2010 to 2022 was taken into account for the literature review. The study analysis has covered all studies, including meta-analysis, case reports, randomized controlled trials, as well as case-control studies and prospective studies. All duplicate studies were removed from the literature, and only recently published studies were selected for the review.

Articles were selected for literature review following the satisfaction of the inclusion and exclusion criteria of the study. The inclusion criteria suggested that all articles selected for the study be published in the English language between the period of 2010 to 2022. Articles published in languages other than English and before 2010 were excluded from the study. The effects of opioids on the cardiovascular system have been of growing interest in the field of medical science. Opioids demonstrate several effects on the cardiovascular system that can be visualized with the help of an ECG [33]. It may also display several changes in the electromyographic films in terms of the progression and regression of the QT interval [30]. The QT interval refers to the time between the beginning of the Q wave and the end of the T wave, which depicts ECG repolarization and ventricular depolarization electrical presentation [34]. QT interval prolongation demonstrates slow electrical conduction of the ventricle [35]. Since the interval of QT changes with the heart rate, a few formulas for the formulation of the heart rate QT interval exist, such as Framingham, Fridericia, and Bazette. Bazette’s formula is considered one of the most common formulas to calculate the QT interval (QTc = QT/√RR) [36]. Here, RR implies the distance among the ridge of two successive R waves. In females, the QTc interval is greater than 470 ms, whereas, in males, it is greater than 450 ms. The rate remains persistent regardless of which formula for correction is used [32]. Moreover, TdP is self-limited; however, it mostly turns into ventricular fibrillation, a life-threatening condition that can lead to death unexpectedly [37].

QT interval prolongation is related to the repolarization of lengthened cardiac events that begin through quick potassium overflow by the channel of cardiac rapid-rectifying [38]. The quick-rectifying cardiac channel is hidden by hERG (KCNH2), and its hampering by a few representators is considered a prominent mechanism for QT interval prolonging [35]. Hence, there exist many fundamental states that can lead substance abuse to the events of QT prolongation, such as HIV infection, cardiac disease, and female gender [39]. A few drugs (antiretroviral protease inhibitors) lead to QT interval prolongation and a greater level of these agents, such as antiretroviral protease inhibitors (IDV, RTV, and ATV), macrolide antibiotics (roxithromycin, erythromycin, dirithromycin, and clarithromycin), antifungal azole agents (itraconazole, ketoconazole, and fluconazole), buprenorphine metabolizing enzyme, and methadone [40]. This literature aspires to examine the impact of opioids accessible on the market on arrhythmogenic susceptibility and QT interval [35].

Patients with QT prolongation are first treated with obstruction, if at all possible. It is important to lower the dose of drugs that lead to QT interval prolongation [41]. Some agents alone may lead to QTc mild prolongation; however, when they are taken in conjunction with other medicines, they impede their metabolism. For example, with cisapride and terfenadine, considerable prolongation can occur. However, when QT prolongation leads to torsade de pointes, it presents an alarming situation [42]. This arrhythmia's clinical manifestations range from cardiac arrest to asymptomatic, self-limiting dysrhythmia [40]. Moreover, it can present clinically as anything from an asymptomatic, self-limiting dysrhythmia to a cardiac arrest [43]. Both non-pharmacological and pharmacological techniques (isoproterenol or magnesium) can be applied for immediate treatment [42]. Increasing heart rate, reducing QT interval, and avoiding premature or aberrant excitability are the main objectives of the treatment [44].

Long-term effects of opioids may result in several reinforcement disorders, such as tolerance, addiction, physical dependence, vomiting, drowsiness, nausea, itching, respiratory depression, increased sensitivity to pain, work disruption, reduced level of attention, hypothermia, hallucinations, urticaria, dizziness, urinary hypotension, delirium, headache, muscle rigidity, and bradycardia [30]. Long-term usage of opioids may also lead to tolerance, which causes adaptations at the neurological level and ultimately reduces the effect of drugs [29]. The tolerance results from its association with several other conditions, such as depression of respiration, urinary retention, and itching [32]. It requires the intake of higher doses to compensate for the effects that cannot be managed by the previous doses of opioids [33]. The second major side effect of opioids is physical dependence, as the individual’s body physiologically adapts to the presence of opioids [34]. However, the withdrawal symptoms include severe irritability, nausea, opiate dose craving, myalgia, vomiting, rhinorrhea, and tremors [30]. Slow reduction of opioid use over days or weeks eliminates the withdrawal symptoms [45]. The severity and speed of drug withdrawal are dependent on the opioid’s half-life. The withdrawal effects of opioids get mediated through medicines such as clonidine [36]. Drug addiction has also been seen to be a defined adverse effect of opioids [32].

The effects of opioids are dependent upon the type of opioid that has been taken into consideration for intake [38]. Opioids are classified as natural opiates, endogenous opiates, esters of morphine, synthetic semisynthetic, and fully synthetic opiates [33]. The endogenous opiates include enkephalins, endorphins, endomorphins, and dynorphins [39]. Opium alkaloids and their derivatives include morphine, thebaine, codeine, and oripavine [30]. Esters of morphine are classified as nicomorphine, diacetylmorphine, dipropanoylmorphine, methyldesophine, desomorphine, diacetyldihydromorphine, and dibenzoylmorphine [37]. Ethers of morphine have further classes of heterocodeine, dihydrocodeine, and ethyl-morphine [31]. Semi-synthetic alkaloid derivatives include oxycodone, hydromorphone, buprenorphine, hydrocodone, oxymorphone, and etorphine. Synthetic opioids consist of anilidopiperidines (alfentanil, remifentanil, fentanyl, sufentanil, carfentanyl, and alpha-methyl fentanyl), benzimidazoles (metonitazene, etonitazepyne, metodesnitazene, etonitazene, clonitazene, and isotonitazene), diphenylpropylamine derivatives (methadone, propoxyphene, dextromoramide, difenoxin, loperamide, dextropropoxyphene, levomethadyl acetate, and piritramide), benzomorphan derivatives (pentazocine, dezocine, and phenazocine), morphinan derivatives (nalbuphine, levomethorphan, butorphanol, levorphanol, and racemethorphan), and oripavine derivatives (dihydroetorphine, buprenorphine, and etorphine) [38].

Buprenorphine is a mu receptor performer that is partially semi-synthetic, which steadily separates the receptor [30]. It can be utilized in OMT for reducing pain since it has an extended duration of impact [45]. Additionally, it is an agonist of the delta receptor, a partial agonist of the nociceptive receptor, and a kappa receptor antagonist [35]. Buprenorphine has a potency between 25 and 100 times greater than that of morphine [30]. The effect of buprenorphine on the QT interval and TdP has been the subject of numerous research, and nearly all of them indicate safe therapy with buprenorphine (3 mg) with no effect on the QT interval [46]. According to a rat study, buprenorphine subcutaneous treatment at doses of 0.006, 0.03, and 0.15 mg/kg reduces QT intervals and accelerates heart rate [32].

At low and medium dosages (0.006, 0.03 mg/kg), but not at high doses (15 mg/kg), this dose-dependent impact is reversible [47]. Buprenorphine/naloxone did not demonstrate a clinically meaningful increase in QT interval whether used alone or in combination with antiretroviral (ARV) drugs [40,41]. None of the 80 OMT patients evaluated by Stallvik et al. who had BMT had a QTc interval longer than 450 ms, and no correlation was found between the patient's QTc intervals and their serum buprenorphine concentrations [48]. According to another study, buprenorphine is a safe substance [40]. Buprenorphine can resolve ventricular arrhythmia and QT prolongation secondary to methadone, which supports the hypothesis that it should be preferred to methadone [42]. Buprenorphine is a safe medicine in this regard since, according to the studies stated above, it does not appear to cause clinically significant QT interval prolongation or arrhythmia when used at typical doses [49].

Methadone, being an agonist synthetic mu receptor, is used to treat pain and OMT [45]. It is a widely used medicine for the management of opioid dependence due to its effectiveness, cost efficiency, and dose flexibility [50]. However, the greater issue is its QT-prolonging impact, which can result in TdP as a potentially fatal arrhythmia, in addition to certain major adverse effects, such as hypotension and oedema [40]. In a study on 2735 patients with a history of using a variety of prescribed medicines, the authors concluded that methadone, as a prescription drug, was most likely to cause TdP. In 2004, ten cases of prolonged QT interval were reported, of which three showed TdP [42]. Two of the patients had ventricular tachycardia, and one of them had experienced a premature ventricular contraction (PVC) bigeminy [49]. Except for one patient, all ten patients were taking medications other than methadone that have pharmacological interactions with methadone, along with oral manual muscle testing (MMT) (dose range: 14-360 mg/day) [45]. More cases of TdP in people taking MMT and other concomitant medications have also been reported [50]. For instance, in three case reports, patients receiving the well-known QT interval-lengthening drugs methadone and cocaine also developed TdP [42].

The primary uses of morphine include the treatment of acute and chronic pain and cancer-related conditions. Another morphine-derived mu receptor agonist is hydromorphone [20]. A few studies have examined how morphine influences the TdP and QT intervals [41]. Fanoe et al. were unable to determine a connection between QT duration and the use of morphine (mean dose = 120 mg, the dose range = 30-300 mg) [35]. In a similar vein, a more recent study investigated the effects of morphine and methadone on mean arterial pressure, heart rate, and interval of QT and found no connection between the two, whereas methadone markedly lengthened the QT interval. They also showed that the QT interval responded to methadone in a dose-dependent manner [42].

Fentanyl, another synthetic mu receptor agonist, is used before surgeries along with cosmetic medications [40]. Fentanyl has minimal effects on the cardiovascular system [32]. All opioids have the side effect of hypotension; however, fentanyl is less problematic than other opioids in this regard [36]. However, the drug, in conjunction with benzodiazepines, may result in decreased stroke volume and cardiac output [35]. One study found that remifentanil and fentanyl do not interfere with the QTc interval, but remifentanil was successful in the reduction of QT interval dispersion, whereas fentanyl failed to halt the increase in QTc and QT dispersion of the interval following intubation [38]. According to one study, premedicated patients with alfentanil reduces the QT interval prolongation brought on by laryngoscopy; however, their effect was not observed in the prolongation of QTc that occurs following the intubation [32]. Overall, it appears that fentanyl and its analogs have a dose-dependent impact on the QT interval [29]. The likelihood of QT interval prolongation is lower at low dosages (5 g/kg), although it may be higher at high doses [25]. Nonetheless, there are no reports of arrhythmia occurring after taking these medications [42].

Although the drug does not show any severe side effects on the heart, the painkiller oxycodone, a mu receptor agonist, can also lead to hypotension and bradycardia. Fanoe et al. conducted the initial study to investigate how oxycodone impacted the QT interval [35]. The authors created a cross-sectional examination of 100 patients who received morphine, tramadol, methadone, and oxycodone treatment for persistent non-malignant pain [35]. Their findings demonstrated that higher doses of oxycodone (more than 100 mg) resulted in a longer QT interval, along with validation of the methadone dose-dependent effect on the QT interval [36]. Another recent study revealed that an oxycodone overdose can result in QT prolongation. For patients with abnormal QT, the median dosage was 100 mg [37]. In addition, a case study revealed TdP in patients receiving large doses of oxycodone following QT interval lengthening [39].

Tramadol is a common pain reliever and a modest artificial mu receptor agonist. An overdose of tramadol can result in nausea, vomiting, tachycardia, respiratory and central nervous system depression, along with agitation and seizures [20]. In a recent study, tramadol administration was associated with a considerable lengthening of the QT interval [21]. Due to harmful side effects, including decreased blood pressure, cardiac output, myocardial contractility following intravenous injection, and central nervous system (CNS) toxicity from prolonged oral usage, meperidine or pethidine, an agonist of the mu receptor, is used less frequently [22]. QTc-interval prolongation is caused by meperidine, which correlates with normeperidine plasma levels, according to a recent study on 58 patients who were administered meperidine with a dose of 304 ±133 [25]. Levorphanol is an antagonist of the N-methyl-D-aspartate receptor and a delta, mu, kappa1, and kappa3 agonist [23]. It is used as an analgesic for patients who do not respond to conventional opioids since it also has serotonin and norepinephrine reuptake inhibitor properties [31].

A major side effect of coronary artery bypass surgery and myocardial infarction is atrial fibrillation, a frequent heart arrhythmia [28]. A link between opioids and atrial fibrillation (AF) has been demonstrated in a few recent investigations. In a study on stroke, the correlation between the prevalence of rising AF and opioid use was explained by the reasons for racial and geographic differences [35]. The most frequently utilized opioids in this study were hydrocodone, propoxyphene, and tramadol. In 2016, a sizable study compared the incidence of AF in patients receiving and not receiving morphine as an analgesic to examine the relationship between morphine and the incidence of AF in Taiwanese breast cancer patients [21]. In this trial, 73,917 individuals were involved, of whom 18,671 received morphine and 55,246 did not. The results depicted that morphine-treated patients had a higher and more noticeable chance of developing AF [32].

## Conclusions

Due to the emergence of the medical field, opioids are also used in the management of pain for non-cancer patients. One of the adverse effects of opioids is a cardiac irregularity in terms of QT prolongation between two successive heartbeats. Despite being well-liked by patients, methadone carries a higher risk of arrhythmias. Tramadol and oxycodone can be categorized as drugs with intermediate risk, whereas morphine and buprenorphine are among the low-risk opioids. If the signs of withdrawal and dependency are recognized earlier, a referral should be ensured to move toward the treatment of addiction. ECG findings suggest that treatment with methadone significantly reduces the interval between QTc among patients with a higher risk of TdP.
